# Supplementary material for: Causal factors in primary open angle glaucoma: a phenome-wide Mendelian randomisation study
Source: Sci Rep. 2023 Jun 20;13:9984. doi: 10.1038/s41598-023-37144-7 (PMC10282034; doi:10.1038/s41598-023-37144-7)
Supplement: Supplementary file 2 — Supplementary Information 2. [file 41598_2023_37144_MOESM2_ESM.docx]

**Supplementary information**

**Legends for supplementary content:**

**Supplementary table 1.1:** A table detailing all traits associated with POAG across all robust measures and in a leave one out analysis. Trait ID = The identifier for this trait within the TwoSampleMR R programme. nSNP = number of SNPs used in analysis. P value for inclusion = the significance threshold at which SNPs were selected for the exposure variable. OR = Odds radio. LCI95 = lower limit of 95% confidence interval. UCI95 = upper limit of 95% confidence interval. LOO = leave one out analysis with MRE IVW indicating whether results were significant throughout a LOO (all_significant) or if there were tests in which the result became insignificant (not_robust). FDR = false discovery rate corrected IVW p value. SE = standard error.

**Supplementary table 1.2:** A table detailing all traits associated with POAG in FDR MRE IVW and leave one out analysis. Note that these traits are not reported in text and have not been manually inspected for sample overlap, although this was done automatically for explicitly UKB identified GWAS studies as detailed in the methods. For this reason, authors interested in the below traits are advised to identify the GWAS used for the exposure variable via TwoSampleMR and examine population overlap in order to ascertain the risk of bias. Trait ID = The identifier for this trait within the TwoSampleMR R programme. nSNP = number of SNPs used in analysis. P value for inclusion = the significance threshold at which SNPs were selected for the exposure variable. OR = Odds radio. LCI95 = lower limit of 95% confidence interval. UCI95 = upper limit of 95% confidence interval. LOO = leave one out analysis with MRE IVW indicating whether results were significant throughout a LOO (all_significant) or if there were tests in which the result became insignificant (not_robust). FDR = false discovery rate corrected IVW p value. SE = standard error.

**Supplementary table 1.3:** A table detailing analysis of the relationship with traits phenome-wide and POAG. Note that these traits are not reported in text and have not been manually inspected for sample overlap, although this was done automatically for explicitly UKB identified GWAS studies as detailed in the methods. For this reason, authors interested in the below traits are advised to identify the GWAS used for the exposure variable via TwoSampleMR and examine population overlap in order to ascertain the risk of bias. Trait ID = The identifier for this trait within the TwoSampleMR R programme. nSNP = number of SNPs used in analysis. P value for inclusion = the significance threshold at which SNPs were selected for the exposure variable. OR = Odds radio. LCI95 = lower limit of 95% confidence interval. UCI95 = upper limit of 95% confidence interval. LOO = leave one out analysis with MRE IVW indicating whether results were significant throughout a LOO (all_significant) or if there were tests in which the result became insignificant (not_robust). FDR = false discovery rate corrected IVW p value. SE = standard error.

**Supplementary table 1.4:** A table detailing MVMR analysis to assess for any confounders in the relationship between 'diabetes diagnosed by doctor' and POAG. BMI = body mass index, BP = blood pressure, LDL = low density lipoprotein, HDL = high density lipoprotein, MVPA = moderate to vigorous physical activity. Correlation = the assumed level of correlation between trait 1 and 2 in the MVMR analysis. We have intentionally selected extremes of high and low levels of correlation between the traits to ensure our results are accurate regardless of the true level of correlation. Note that both ‘Trait 1’ and ‘Trait 2’ are insignificant across all analysis, which means it is not possible to determine if the relationship between diabetes and POAG is underpinned by a confounding variable.
